# Precordial pain induced by the isolated cardiac hydatid cyst in interventricular septum: a case report

**DOI:** 10.1186/s13019-023-02247-9

**Published:** 2023-05-06

**Authors:** Tiange Li, Yunfei Ling, Yongjun Qian

**Affiliations:** Department of Cardiovascular Surgery, West China Hospital, Sichuan University, Guoxuexiang 37th, Chengdu, 610041 Sichuan P. R. China

**Keywords:** Cardiac hydatid cyst, Interventricular septum, *Echinococcus granulosus*

## Abstract

**Background:**

Human hydatid disease occurs after infection with *Echinococcus granulosus*, mainly involves liver and lung, while hydatid involves heart is infrequent. A great majority of hydatid diseases could be asymptomatic, and incidentally found through examination. Here, we reported a woman who suffered an isolated cardiac hydatid cyst located at the interventricular septum.

**Case presentation:**

A 48-year-old woman presented intermittent chest pain was admitted to the hospital. Imaging examination revealed a cyst located at the interventricular septum near the right ventricular apex. Considering medical history, radiological findings and serological results, cardiac hydatid disease was suspected. The cyst was successfully removed, while pathological biopsy confirmed the diagnosis of infection of *Echinococcus granulosus*. Postoperative course was uneventful, the patient was discharged from hospital without complications.

**Conclusion:**

For symptomatic cardiac hydatid cyst, surgical resection is necessary to avoid progression of disease. During surgical procedure, appropriate methods to reduce the potential risk of hydatid cyst metastasis are essential. Besides surgery, combined with regular drug therapy is an effective strategy to prevent reappearance.

## Background

Human hydatid disease (HD) is a parasitic infection disease, occurs mainly as a result of infection with the larvae of *Echinococcus granulosus*. Dogs are main primary hosts, while sheep are intermediate hosts, thus, HD most commonly occurs in people who raise sheep. The most commonly involved organ of hydatid cysts (HC) is the liver, while cardiac involvement is uncommon [1]. A considerable proportion of the HD patients can stay asymptomatic, until the space-occupying effects appear on the involved organs [2]. However, patients with cardiac HC usually appear symptomatic, though the exact proportion remains unknown [3, 4]. Chest pain, dyspnea, syncope and palpitations are common symptoms in patients with cardiac HC, while patients can also show infection-associated symptoms such as cough and fever [1, 5]. Among all cases of cardiac HC, the interventricular septum (IVS) is the site rarely involved [1, 2, 5]. Here we reported a rare case of isolated cardiac hydatid cyst located at IVS, meanwhile, without any other organs involvement.

## Case presentation

A 48-year-old female from Tibet, China was admitted to our hospital for a 4-month history of intermittent pain of chest and back. Each time the pain attacked would last about 1 h and usually twice a day, and it often followed with mild fever and headache. The patient engaged in sheep and cattle raising, without any other heart diseases had occurred before. Electrocardiography demonstrated sinus bradycardia at a rate of 47 beats per minute without ST segment or T wave changes, either conduction abnormality. Cardiac murmur was not found within each cardiac cycle. For routine laboratory tests, all results remained in the normal limits, except an elevated percentage of eosinophils (6.3%).

**Fig. 1:**
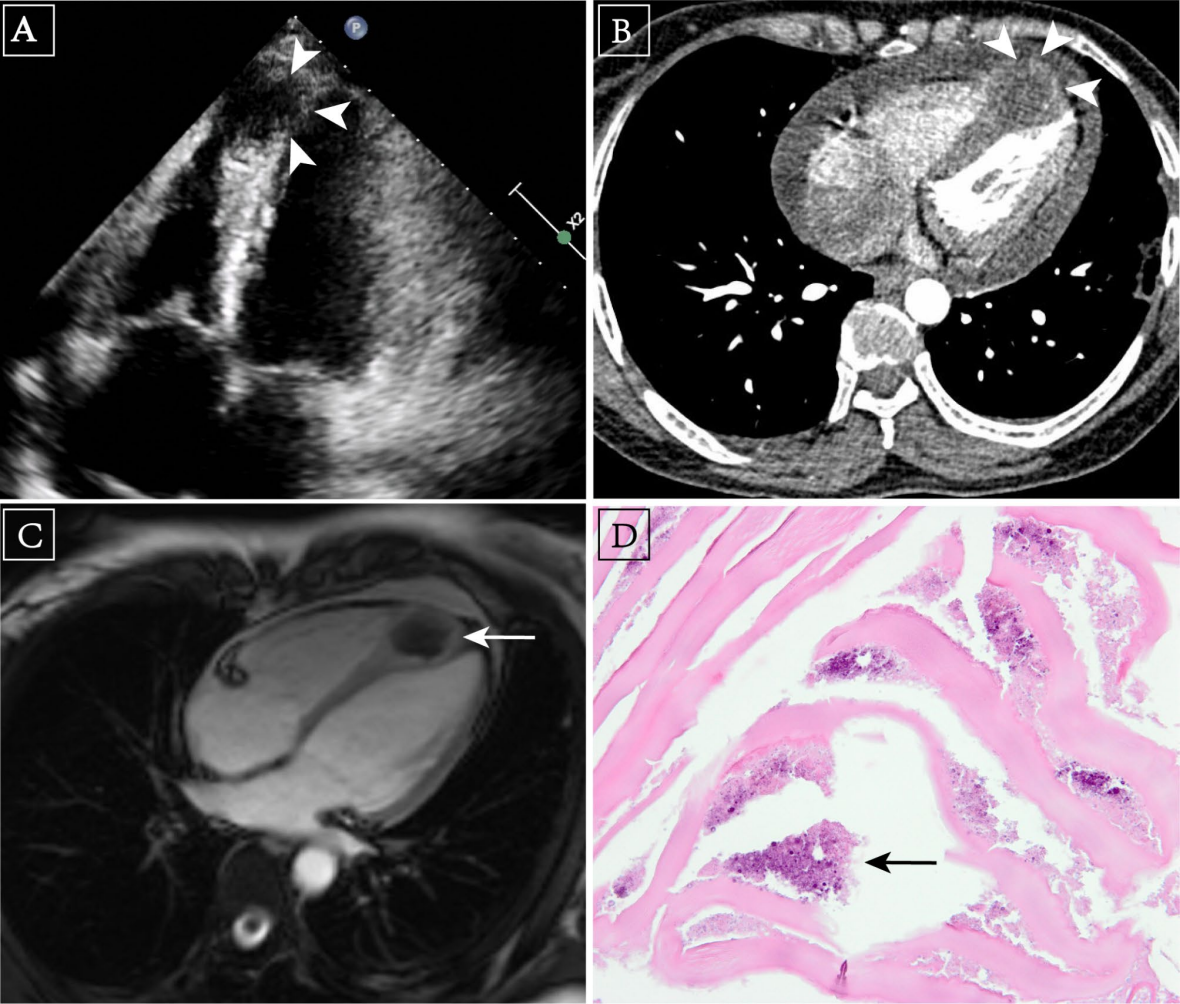
** A**, Four chambers section of apex in transthoracic echocardiography image. An echocardiography demonstrated a weak-echo nodule located at the myocardium of the right ventricular apex (arrowhead). **B**, Cardiac hydatid cyst in CT image. A contrast-enhanced CT revealed a soft-tissue nodule within the interventricular septum near right ventricular apex without significant enhancement (arrowhead). **C**, Cardiac hydatid in MRI image. A contrast-enhanced MRI demonstrated no significant enhancement of the mass (arrow), while myocardium was significantly enhanced. **D**, Pathological image of excisional hydatid cyst (H&E, arrow)

Chest radiography showed no lung parenchymal abnormality, either no pleural effusion. Among further cardiac examination, the main anomaly found by transthoracic echocardiography (TTE) was a slightly weak-echo mass located at the myocardium of the right ventricular apex (Fig. 1, *panel A*). TTE revealed normal cardiac function. Moreover, a small patent foramen ovale, mild tricuspid regurgitation and pericardial effusion were revealed. Computed tomography angiography (CTA) revealed a soft tissue nodule without enhancement located in the IVS near the right ventricular apex, the boundaries between the mass and surrounding myocardium were indistinct (Fig. 1, *panel B*). To clarify the diagnosis, enhanced MRI of the heart was carried out and revealed a cystic, heterogeneous-intensity IVS mass (3.0 cm× 2.8 cm) which was isointensity in T1-weighted images, slight hyperintensity in T2-weighted images, and no significant enhancement in contrast-enhanced images (Fig. 1, *panel C*). Imaging characteristics of CTA and MRI suggested the possibility of cardiac HD [6, 7], thus, further antibody assay of parasite was performed with a positive result of *Echinococcus granulosus* antibody.

Given the radiological findings and clinical features, we recommended the patient received surgery to excise the cyst. The patient consented to surgical treatment and signed the operation agreement. During the operation, a median sternotomy was performed, then cardiopulmonary bypass (CPB) was initiated through aortic and bi-caval cannulation. The patient was anticoagulated with 1 mg/kg heparin administered intravenously, while antegrade cardioplegia was administrated to induce cardiac arrest. A mass could be seen at the apex of the heart. Then, we performed regular right atriotomy, and the cyst was exposed through the atrium incision and tricuspid orifice. After puncturing and drawing the cyst content, 3% hypertonic saline was injected into the cyst, and then, excision of the cyst was done. Physiological saline was injected into the residual cavity to verify the presence or absence of ventricular shunt. After that, the residual cavity was plicated with continuous sutures. At last, closed the right atrium, and poured 3% hypertonic saline into the pericardial cavity to prevent local dissemination. Intraoperative TEE showed no mitral or tricuspid regurgitation, either no residual shunt on the IVS, Histopathological examination confirmed the diagnosis of cardiac HC (Fig. 1, *panel D*). Albendazole, with the dose of 400 mg twice daily, was told to be regularly used for 24 weeks postoperatively to prevent recurrence and metastasis of hydatid. Postoperative vital signs were stable and the patient was discharged home without symptom of chest pain and other complications.

## Discussion

*Echinococcus granulosus* is a helminth parasite, dogs and other carnivorous animals are primary hosts and sheep are intermediate hosts, humans are usually infected as intermediate accidental hosts, which causes echinococcosis or HD. Humans are usually infected through ingesting food, milk, or water contaminated by dog feces containing the ova of the parasite [1]. The most common sites of hydatid cysts (HC) are the liver, and then are lungs [2], only 0.5–2% HD patients show a cardiac involvement [3–5, 8, 9], such a low incidence possibly attributes to persistent movement and contraction of myocardium [10]. The distribution of cysts in the heart depends on the coronary blood supply, the left ventricular wall is the most common cardiac location, followed by the right ventricle, pericardium, left atrium, and right atrium [1, 7, 11]. The interventricular septum (IVS) is less frequently involved, just reported in 4% of all cardiac cases [2]. However, in the majority cases of cardiac HC, the disease usually affects other organs simultaneously [12], in other words, cardiac HC is more likely to happen secondary to hepatic HC or pulmonary HC. The case we reported, HC located at the IVS without imaging findings of liver or lung involvement, whereas, is extremely rare.

Most of HD patients can remain asymptomatic for many years, whereas, the condition seems different in patients with cardiac HC. S. Fennira and colleagues reviewed cases of HC in the IVS from 1964 to 2019, finally included 45 cases, showed only 5 patients (11%) were asymptomatic [1]. Yaman ND also reviewed studies of cardiac *echinococcosis* worldwide, which included 86 patients, only 5 patients (6%) were asymptomatic. These findings suggest cardiac HC is a more serious condition and easier to present clinical symptoms. The type and severity of clinical manifestations mainly depend on the organ involved, the number and size of the cyst and other complications [6], thus, symptoms are various. Compression to coronary arteries by a cyst can cause myocardial ischemia, easily give rise to precordial pain, and more severe, it may cause myocardial infarction, which increase the incidence of sudden death. If the cyst gives the compression to the cardiac conduction system, conduction block will happen. If it gives the compression to pulmonary artery, dyspnea and cyanosis may occur [8]. When the cyst has a tendency of intracardiac development, direct mechanical interference to valves and changes to the size of chambers will influence the cardiac function, results in symptoms of heart failure, such as dyspnea, weakness, dizzy and edema. Complications including bacterial infection, cyst rupture and most serious, anaphylactic shock. However, exact prevalence remains unclear. This patient presented with intermittent chest pain, which suggested acute myocardial ischemia.

Some serological tests such as eosinophil count, indirect hemagglutin, enzyme-linked immune sorbent assay and Casoni intradermal test have clinical diagnosis value for *Echinococcus granulosus* infection, but due to the false negativity and limited sensitivity and specificity, are usually not enough to confirm diagnosis. If histopathological examination is unavailable, the diagnosis is primarily confirmed by combination of clinical findings, imaging and serology. Eugenio Zalaquett and colleagues concluded features of HD on ultrasound, CT and MR imagings, and classified HC into five types to help clinical diagnosis [6]. Final diagnosis should be confirmed by histopathological examination, different developmental stage of *Echinococcus granulosus* could be found. Aviral Gupta reported a case of cardiac HC, showed a pathological image of acellular lamellated membranes of HC with partially autolyzed brood capsules, which confirmed the diagnosis [13]. Similar HC in the background of myocardium could also be found on our pathological image.

Cause there is still no specific medicine for cardiac HC, surgical excision is the preferred treatment, and it should not be delayed, since the occurrence of cyst rupture, anaphylactic shock and other potentially lethal complications cannot be accurately predicted [1]. Selection of surgical procedures are depended on the location, size and type of the cyst. Vivek Wadhawa reported a case series, which included 10 patients with cardiac HC, 9 patients underwent cystectomy under CPB, only one patient with multiple cysts with pericardial involvement underwent cystectomy without CPB [14]. For most patients with cysts in the IVS, cystectomy underwent CPB are the safest way [1]. The principles of surgery is complete excision of germinal layer, which is the basement to achieve radical cure [2]. Preparing and applying helminthicide and avoiding contamination of the surgical field are extremely important, reported helminthicide includ 20% hyper tonic saline solution, 1% iodine solution, 2% formalin, 0.5% silver nitrate solution, or 5% cetrimonium bromide solution [1, 15]. While Vivek Wadhawa recommended 3% hypertonic saline solution as an effective and nontoxic helminthicide in order to prevent dissemination during operation [14], which is in accordance with our habits. In a word, surgical management of cardiac HC in the IVS is challenging, multiple risk factors should be considered when the choice of surgical procedure is made.

## Conclusion

We described a rare case of cardiac HC located at the IVS without liver or lung involvement. Surgical excision was successfully performed, while pathological image confirmed the diagnosis of cardiac HC. The case highlights the importance of imaging examination for unexplained chest pain, while surgical excision is necessary for symptomatic HD patient. Besides, to avoid metastasis and reappearance, surgical procedures should be completely and carefully.

## Data Availability

Data sharing is not applicable to this article as no datasets were generated or analyzed during the current study.

## Abbreviations

HD: hydatid disease
HC: hydatid cyst
IVS: interventricular septum
TTE: transthoracic echocardiography
CTA: computed tomography angiography.
